# Frequent Spindle Assembly Errors Require Structural Rearrangement to Complete Meiosis in *Zea mays*

**DOI:** 10.3390/ijms23084293

**Published:** 2022-04-13

**Authors:** Jodi D. Weiss, Shelby L. McVey, Sarah E. Stinebaugh, Caroline F. Sullivan, R. Kelly Dawe, Natalie J. Nannas

**Affiliations:** 1 Department of Biology, Hamilton College, Clinton, NY 13323, USA; jodi.weiss.jw@gmail.com (J.D.W.); smcvey@hamilton.edu (S.L.M.); sstinebaugh718@gmail.com (S.E.S.); carolinefsullivan@gmail.com (C.F.S.); 2 Department of Genetics, University of Georgia, Athens, GA 30602, USA; kdawe@uga.edu; 3 Department of Plant Biology, University of Georgia, Athens, GA 30602, USA

**Keywords:** spindle, error correction, meiosis, chromosome

## Abstract

The success of an organism is contingent upon its ability to faithfully pass on its genetic material. In the meiosis of many species, the process of chromosome segregation requires that bipolar spindles be formed without the aid of dedicated microtubule organizing centers, such as centrosomes. Here, we describe detailed analyses of acentrosomal spindle assembly and disassembly in time-lapse images, from live meiotic cells of *Zea mays*. Microtubules organized on the nuclear envelope with a perinuclear ring structure until nuclear envelope breakdown, at which point microtubules began bundling into a bipolar form. However, the process and timing of spindle assembly was highly variable, with frequent assembly errors in both meiosis I and II. Approximately 61% of cells formed incorrect spindle morphologies, with the most prevalent being tripolar spindles. The erroneous spindles were actively rearranged to bipolar through a coalescence of poles before proceeding to anaphase. Spindle disassembly occurred as a two-state process with a slow depolymerization, followed by a quick collapse. The results demonstrate that maize meiosis I and II spindle assembly is remarkably fluid in the early assembly stages, but otherwise proceeds through a predictable series of events.

## 1. Introduction

The faithful segregation of chromosomes during cell division is critical for an organism’s viability and fertility. Mistakes in this process lead to aneuploidy [1], which is associated with tumorigenesis and metastasis in mitotic cells [2,3]. Aneuploidy in meiosis, the specialized cell division that produces haploid gametes, is the leading cause of congenital birth defects and miscarriage [4]. Eukaryotic chromosomes are segregated in mitosis and meiosis by the spindle, a cytoskeletal structure composed of microtubules that polymerize dynamically with the addition of α- and β-tubulin monomers. Spindle microtubules are organized into bipolar arrays with their minus ends clustered at the poles and their plus ends reaching into the midzone, where they attach to chromosomes [5]. Assembly of a bipolar spindle is critical for accurate chromosome segregation. Errors in the assembly process can lead to aberrant spindle structures, such as multipolar spindles, which are often seen in cancer cells [6], and the inhibition of spindle assembly dynamics with small molecule inhibitors leads to severe chromosome mis-segregation [7]. In studies of live human oocytes, unstable meiosis I spindles with multipolar or apolar morphologies yielded high rates of segregation defects as well [8].

Spindle assembly in mitotic and male meiotic animal cells is mediated by centrosomes, which are specialized microtubule organizing centers (MTOCs) localized in the cytoplasm [9]. After the S phase, duplicated centrosomes migrate to opposite sides of the cells, where they nucleate microtubules, and establish a bipolar spindle by polymerizing outward to attach chromosomes in a “search and capture” mechanism [10]. However, not all cell types and species possess centrosomes; female meiotic cells in humans and other mammals [11], *C. elegans* [12], *Drosophila* [13], *Xenopus* [14] and most plants [15] lack these structures. It is likely that the lack of centrosomes makes the spindle assembly process more error prone in the early stages [8,16,17,18]. Studies on live human oocytes showed that more than 80% of cells initially formed multipolar or apolar spindles that required correction [8], and spindle assembly in mouse oocytes requires the active fragmentation and rearrangement of MTOCs into two poles [16,17]. Despite this evidence suggesting a more error-prone process, the mechanisms of acentrosomal spindle assembly are still not as well understood.

In most female animal meiosis, spindles are self-assembled after nuclear envelope breakdown (NEBD), through microtubule nucleation near chromosomes and at dispersed cytoplasmic MTOCs [9,19,20,21]. These cytoplasmic MTOCs are small asters, comprising a γ-tubulin, a specialized version of tubulin monomers that promote radial growth of microtubules [22]. Human oocytes appear to use only chromatin-based nucleation, as spindle assembly initiates solely from asters located within the chromosome mass [8]. Microtubule nucleation near chromatin has been shown to be regulated by the small GTPase Ran and its associated pathway, as well as the Chromosome Passenger Complex, which includes the Aurora B kinase, known for its role in monitoring chromosome attachments to the spindle [23]. With the help of microtubule-associated proteins (MAPs), nucleated microtubules are rearranged and bundled, orienting minus ends towards poles in a “slide and cluster” mechanism [24]. Acentrosomal spindles are thought to have a more fluid structure than their centrosomal counterparts, since their assembly relies on restructuring microtubules into an ordered array [24].

Plants lack centrosomes entirely; all cell divisions, including mitosis, female meiosis and male meiosis, occur with acentrosomal spindles [25,26]. Data from maize [27] and *Haemanthus* [28,29] suggest that the plant acentrosomal assembly process may be different from animals, with microtubules initially organizing on the nuclear envelope, then bundling among the chromosomes after NEBD. Most of our understanding of plant spindle assembly dynamics come from studies of live mitosis in maize [30,31], *Arabidopsis* [32], cultured tobacco cells [33] and the moss *P. patens* [34]. Live imaging of plant meiosis has been more limited, with studies focusing on chromosome dynamics, including rapid movements in prophase I [35,36] or segregation in anaphase I and II [37,38,39], rather than spindle dynamics. Understanding meiotic-specific spindle assembly is critical, as fixed studies have shown that spindle structure can be dramatically different between meiosis and mitosis. Maize, for example, has a narrow meiotic spindle, with highly focused poles compared to the broad, barrel-shape of mitotic spindles [27,40].

A recent study by Prusicki et al. established a live meiotic imaging system in *Arabidopsis* that followed cells from meiotic entry to the final gametes, using fluorescently tagged microtubules (β-tubulin-RFP) and chromatin (Rec8-GFP, a meiosis-specific cohesin component) [41]. By tracking the phenotypic changes of five cellular parameters, including cell shape, microtubule arrays, nucleus and nucleolus position and chromatin condensation, they defined 11 stages or “meiotic landmarks”. They demonstrated that the landmarks could be used to characterize *Arabidopsis* meiotic mutants and their dynamic phenotypes not captured with fixed imaging. We sought to establish a similar understanding of meiotic progression in maize, given its importance as a model system for meiosis [42] and the large number of identified meiotic mutants whose molecular functions remain uncharacterized [42,43,44]. While Prusicki et al. observed the entire meiotic progression, we focused on capturing spindle dynamics with increased spatial and temporal resolution. A recent study of fixed specimens by Xue et al. suggests this process is highly error prone, similar to female animal meiosis [18]. They identified multipolar spindles in wild-type rice, maize, tomato and *Arabidopsis* cells, and concluded active correction must occur because the final meiotic products did not display phenotypic errors [18]. However, these presumed correction events have yet to be directly observed.

Here, we used a live-cell imaging approach [38,39] to visualize maize meiotic spindle assembly in real time. The data confirm the observations of Xue et al. (2019), by demonstrating a high incidence of multipolar spindles in both meiosis I and II that are actively corrected to bipolar form before proceeding to anaphase. Assembly error rates were similar to animal meiosis [8,20], but the assembly process was significantly faster, with a time scale of minutes compared to hours. Consistent with prior results [27,29,32,41,45,46], we find that spindle assembly begins with a perinuclear ring of microtubules on the nuclear envelope that collapses into nuclear space upon NEBD and organizes into a bipolar spindle. We extended our observations to include the spindle disassembly, and found a two-state process, with a switch from slow depolymerization to fast collapse, whereas the phragmoplast, the microtubule-based plant cytokinesis structure [47], expanded at a constant rate. We observed structural fluidity through the correction of many erroneous morphologies, including apolar, tripolar and multipolar spindles; in one instance, two independent spindles fused within a cell. Our observations also showed that meiosis I and II spindle dynamics are indistinguishable and, thus, error correction is inherent to the spindle assembly process.

## 2. Results

We previously demonstrated a method for imaging live maize meiotic cells undergoing chromosome segregation using CFP-β-tubulin to label microtubules and SYT012-labelled chromosomes [38,39]. Here, we used the imaging method to investigate the dynamics of wild-type meiotic spindle assembly and disassembly. We previously confirmed that the CFP-tubulin (β-TUB1) tag does not affect spindle dynamics, nor are the isolated meiotic cells disrupted or compressed by imaging conditions [38]. Live cells were imaged over time in three dimensions. A total of 75 cells (55 meiosis I and 20 meiosis II cells) were imaged undergoing spindle assembly and 76 cells (62 meiosis I and 14 meiosis II cells) were imaged disassembling the spindle and establishing a phragmoplast. All quantification and analysis presented below is based on these live cells. Example movies of spindle assembly (Supplemental Movies S1 and S2), chromosome congression (Supplemental Movie S3), error correction (Supplemental Movies S6–S10), disassembly (Supplemental Movies S4 and S5), and the full process (Supplemental Movies S11 and S12) can be found in the Supplemental Materials.

### 2.1. Spindle Assembly Dynamics in Male Meiosis I and Meiosis II

**Figure 1:**
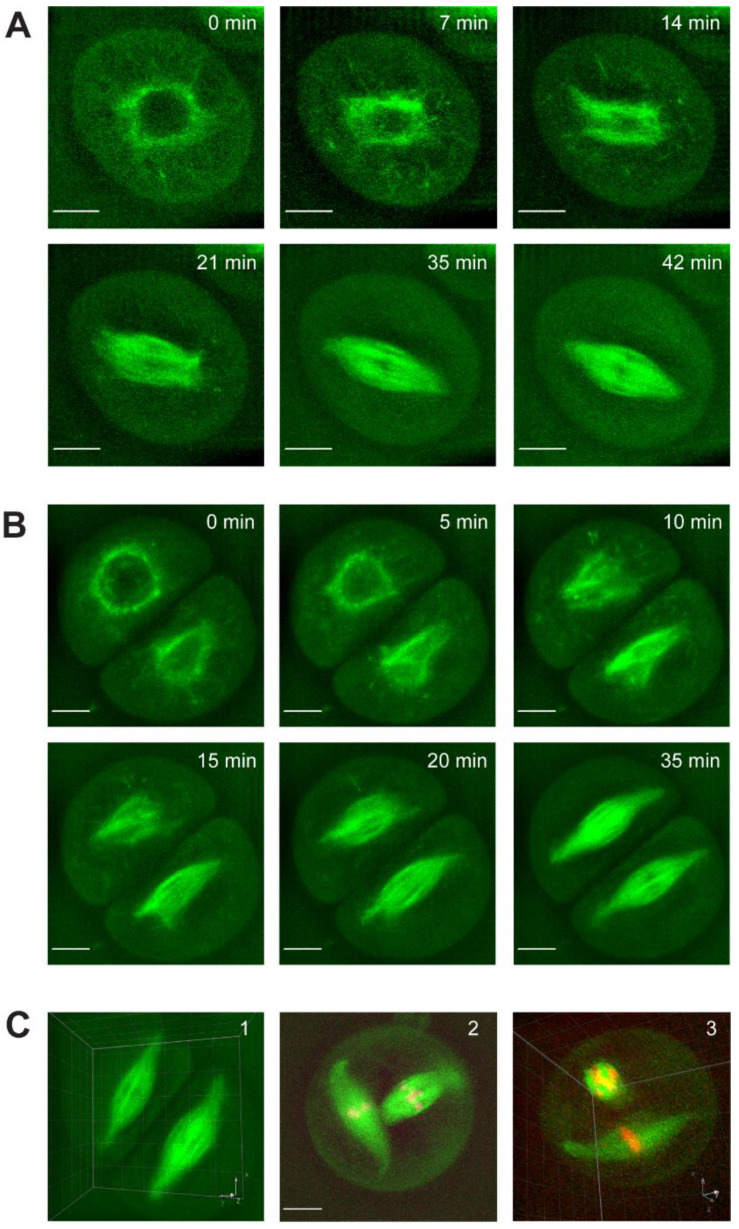
Live assembly of male maize meiosis I and meiosis II spindles. Live assembly of meiotic spindles were imaged using CFP-tubulin (β-TUB1) to label microtubules (green). The cell volume can be seen due to visible diffuse unincorporated CFP-tubulin monomers in the cytoplasm. (**A**) Example movie of meiosis I spindle assembly. (**B**) Example movie of meiosis II spindle assembly. (**C**) A single spindle forms in each meiosis II cell, but assembly results in spindles oriented parallel to each other (panel 1). Of all imaged cells (*n* = 75 total), only a single instance of non-parallel meiosis II spindles was observed (panel 2). Viewed in 3D, the spindles can be seen in a perpendicular orientation (panel 3); a cross-sectional view of the top spindle is seen in panel 3 as the 3D orientation is looking down the length of the spindle. Chromosomes are shown in magenta in panel 2 and Scale bars: 10 µm.

**Figure 2:**
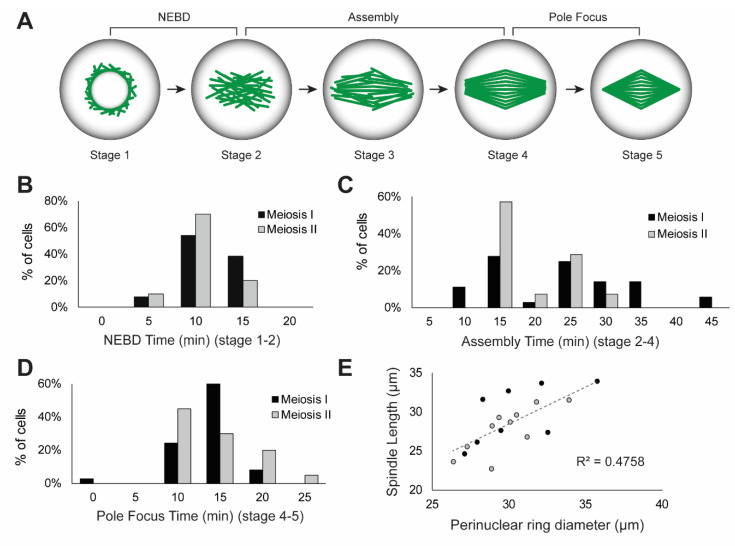
Dynamics of meiotic spindle assembly. (**A**) Meiotic spindle assembly is grouped into five stages; microtubules are shown in green and cell outline in black. Stage 1: nuclear envelope with surrounding microtubules. Nuclear envelope breakdown (NEBD) occurs in the transition from stage 1 to stage 2: collapse of microtubules into the nuclear space. Spindle assembly occurs from stage 2 through stage In stage 3, a spindle shape is emerging and by stage 4, a bipolar spindle with broad poles is visible. From stage 4 to the final stage 5 (fully formed bipolar spindle), the poles are focused to tight points. (**B**) Histogram of the NEBD time (stage 1–2) in meiosis I (black) and meiosis II (gray) cells. (**C**) Histogram of spindle assembly time (stage 2–4) in meiosis I and meiosis II cells. (**D**) Histogram of spindle pole focusing time (stage 4–5). (**E**) Linear correlation of spindle length with perinuclear ring diameter in meiosis I (black) and meiosis II (gray) cells (*n* = 18).

In both meiosis I and II cells, the nuclear envelope collapses, the spindle assembles in the former nuclear space, and the poles become tightly focused (Figure 1A,B, Supplemental Movies S1 and S2). The process of spindle assembly was categorized into five stages to assist quantification (Figure 2A). Microtubules are visible on the periphery of the nuclear envelope in prophase of both meiosis I (Figure 1A, T = 0 min) and meiosis II (Figure 1B, T = 0 min), and extend radially into the cytoplasm. This structure has been previously identified in multiple species as a perinuclear microtubule ring [27,32,33,34,48]. Upon nuclear envelope breakdown (NEBD), microtubule bundles collapse into the nuclear space (Figure 1A, T = 7 min; Figure 1B, T = 0 and 5 min bottom cell, T = 5 and 10 min top cell). NEBD time was measured as the time from intact nuclear envelope (stage 1) to fully collapsed microtubules in the nuclear space (stage 2). Average NEBD time for meiosis I was 10.6 ± 3.3 min and 9.4 ± 3.0 min for meiosis II (no statistically significant difference, *p*-value > 0.05, *t*-test). Meiosis I and II cells have a similar distribution of NEBD times, with no difference in variation (Figure 2B).

Spindles assemble in the nuclear space, initially creating a barrel shape with irregular microtubule structure (stage 3, Figure 1A T = 14, Figure 1B top cell T = 10–15min, bottom cell T = 5–10 min) that is organized into parallel microtubule bundles with a bipolar form (stage 4, Figure 1A T = 21, Figure 1B top cell T = 20 min, bottom cell T = 15 min). The time from collapsed microtubules (stage 2) to bipolar spindle (stage 4) was considered the spindle assembly time (Figure 2A). The average assembly time for meiosis I was 22.9 ± 9.6 min and 18.9 ± 5.5 min for meiosis II. While these averages are not statistically different, with a 95% confidence level cut-off (*p*-value = 0.06, *t*-test), the variance in assembly time is statistically different, with a greater variance in meiosis I assembly time (*p*-value = 0.03, f-test). The variance can be seen in the histogram plot (Figure 2C), where meiosis I has a broader distribution of assembly times than meiosis II, which has a defined peak at 15 min.

After creating a bipolar form, the spindle poles focus into tight points (stage 4–5). The focusing of the spindle poles is a uniquely meiotic feature, as mitotic plant spindles remain barrel shaped throughout chromosome segregation [28,30,32,45,49]. The average pole focusing time was 13.2 ± 3.9 min for meiosis I and 13.7 ± 4.6 min for meiosis II (no statistical difference, *p*-value > 0.05, *t*-test), and they have a similar distribution of pole focusing time, with no difference in variation (Figure 2D).

Total spindle assembly time was defined as the time from NEBD to a bipolar spindle with focused poles (stage 1–5). Total spindle assembly time was the same in meiosis I (46.7 ± 9.8 min) and meiosis II cells (41.9 ± 8.1 min) (not statistically different, *p*-value > 0.05, *t*-test) (Figure 2E). A single spindle formed inside each cell, and in paired meiosis II cells, spindles formed in a parallel configuration (Figure 1C, panel 1). Of all imaged cells (*n* = 75 total), only a single instance of non-parallel meiosis II spindles was observed (panel 2). The perpendicular orientation can be seen with a cross-sectional view of the top spindle (panel 3). Spindles did not elongate during assembly. Instead, microtubules within the perinuclear ring appear to shuffle their arrangement into a bipolar spindle. The length of the assembled spindle correlates with the diameter of the perinuclear ring (R^2^ = 0.4758), with spindle length approximately equal to the diameter of the ring (spindle length is slightly shorter, on average 94.8% of ring diameter) (Figure 2E).

### 2.2. Meiotic Chromosome Congression Dynamics

**Figure 3:**
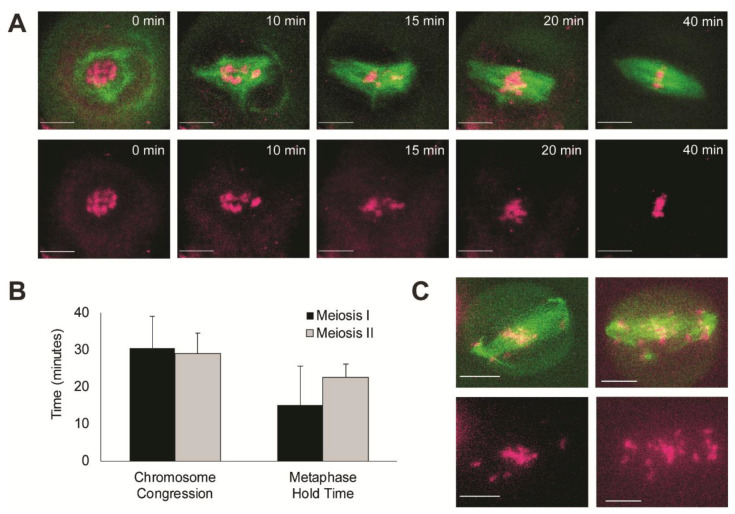
Dynamics of chromosome congression and alignment on the spindle. (**A**) Example movie of chromosome congression dynamics during spindle assembly; chromatin (magenta) labelled via SYTOTop panel: overlay microtubules (green) and chromosomes (magenta); bottom panel: chromosomes alone. (**B**) Average chromosome congression time and metaphase hold time; error bars represent standard deviation. (**C**) Example images of failed chromosome congression; congression failed in 3 of 63 cells. Scale bars: 10 µm.

During spindle assembly, chromosomes congress and align on the forming spindle (Figure 3, Supplemental Movie S3). After NEBD, chromosomes previously contained within the nucleus (T = 0 min) are unrestrained and individual chromosomes can be resolved (T = 10 min). Chromosomes congress to the midzone of the forming spindle (T = 10–40 min), where they display a tight alignment at the spindle equator until the metaphase-to-anaphase transition. Average chromosome congression time is 30.4 ± 8.7 min for meiosis I and 29.0 ± 5.5 min for meiosis II (no statistical difference, *p*-value > 0.05, *t*-test). Once spindle assembly is complete and chromosomes have congressed to the equator, there is a pause in metaphase before the onset of anaphase. This metaphase hold time is 15.0 ± 10.5 min for meiosis I and 22.5 ± 3.5 min for meiosis II (no statistical difference, *p*-value > 0.05, *t*-test) (Figure 3B). Congression also occasionally failed under our culture conditions (*n* = 3/63 observed cells) and individual chromosomes could be seen near spindle poles (Figure 3C). Cells with misaligned chromosomes did not proceed to anaphase.

### 2.3. Spindle Disassembly Dynamics and Phragmoplast Expansion in Meiosis I and II

**Figure 4:**
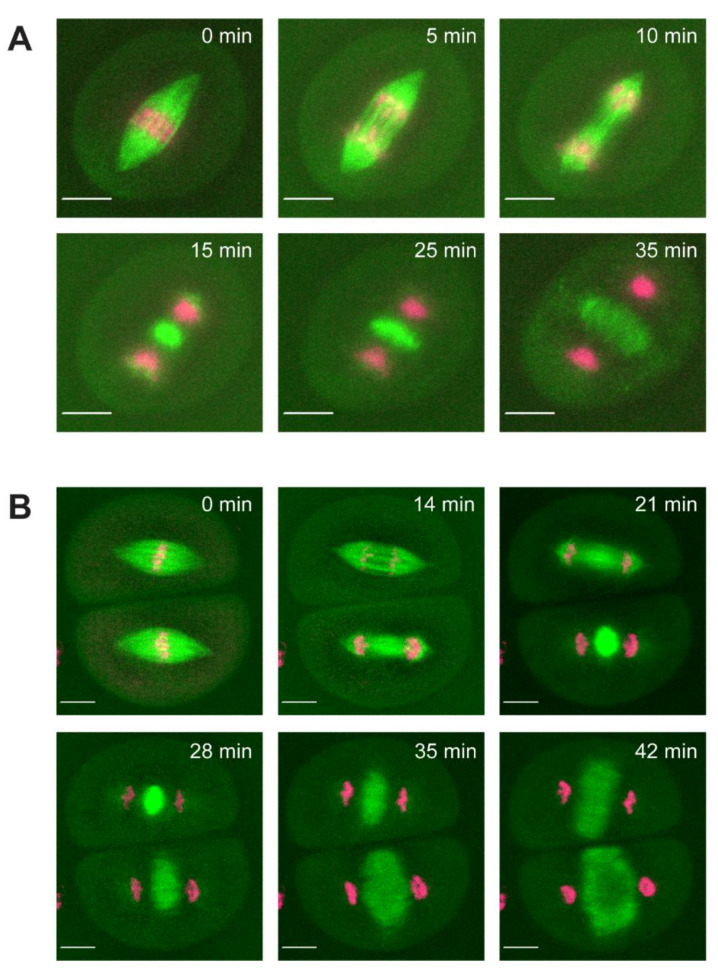
Live disassembly of meiotic spindles and phragmoplast formation. Disassembly of meiotic spindles and the formation of the phragmoplast was imaged using CFP-tubulin (β-TUB1) to label microtubules (green) and SYTO12 to label chromosomes (magenta). (**A**) Example movie of meiosis I spindle disassembly (T = 0–15 min) and phragmoplast expansion (T = 15–35 min). (**B**) Example movie of meiosis II spindle disassembly (0–10 min) and phragmoplast formation (10–35 min). Scale bars: 10 µm.

**Figure 5:**
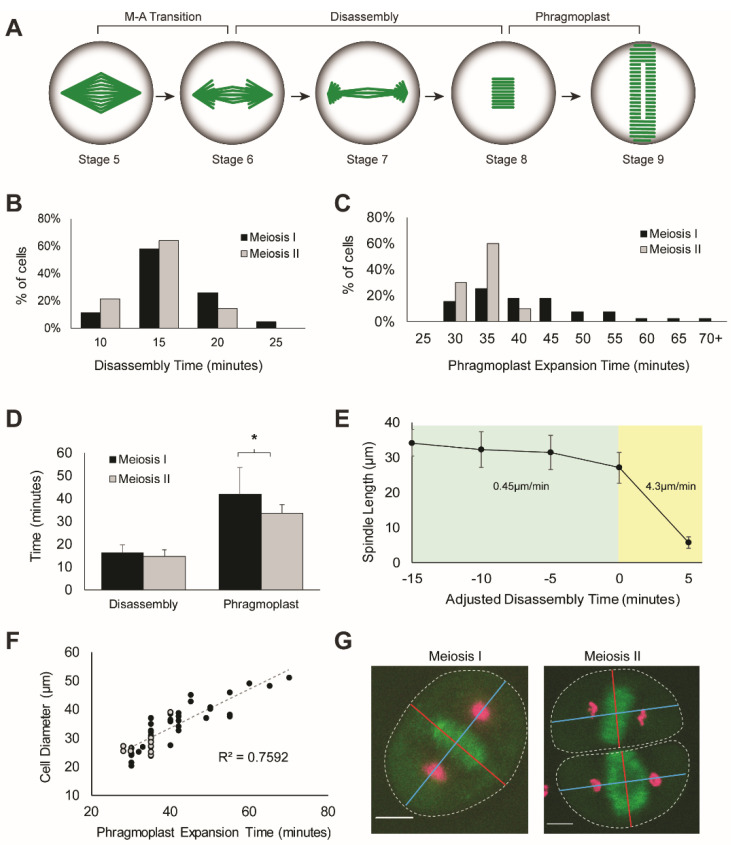
Dynamics of meiotic spindle disassembly and phragmoplast expansion. (**A**) Meiotic spindle disassembly and phragmoplast expansion is grouped into four stages. Metaphase cells (stage 5) undergo the metaphase-to-anaphase transition (M-A transition) and begin depolymerization of kinetochore microtubules (stage 6) to pull chromosomes apart. Kinetochore microtubules are fully disassembled when chromosomes reach the spindle poles (stage 7) and disassembly concludes when interpolar microtubules disappear (stage 8). Phragmoplast formation begins with a nucleating disk (stage 8) that expands radially outward toward the cell cortex (stage 9). (**B**) Histogram of spindle disassembly times (stage 6–8) in meiosis I (black) and meiosis II (gray) cells. (**C**) Histogram of phragmoplast expansion time (stage 8–9) in meiosis I (black) and meiosis II (gray) cells. (**D**) The average disassembly time and phragmoplast expansion time; error bars represent standard deviation. * denotes *p*-value < 0.001, *t*-test (**E**) Spindle length plotted through disassembly time; time is adjusted with T = 0 representing the inflection point between slow (green) and fast (yellow) depolymerization phase (yellow). Displayed depolymerization rates are the calculated slope of each phase; error bars represent standard deviation. (**F**) Linear correlation of cell diameter and phragmoplast expansion time. R^2^ value calculated for total cell population of meiosis I (black) and meiosis II (gray) cells (*n* = 47). (**G**) Phragmoplasts expansion axis (red line) is perpendicular to former spindle axis (blue); correlation plot in (**F**) uses phragmoplast-oriented cell diameter (red line). Scale bar: 10µm.

After successful chromosome alignment in the metaphase, spindles mediate chromosome segregation in anaphase. The dynamics of anaphase chromosome movement were previously analyzed [38], and here, we focused on the microtubule-based dynamics of spindle disassembly and phragmoplast expansion. Example movies of meiosis I and II disassembly and phragmoplast expansion are shown in Figure 4 (Supplemental Movies S4 and S5); the process was categorized into four stages (Figure 5A). Disassembly begins at the transition from metaphase (stage 5, Figure 4A,B T = 0–5 min) to the onset of anaphase (stage 6, Figure 4A T = 5 min, Figure 4B T = 5 min, top cell), characterized by depolymerization of kinetochore microtubules. Kinetochore microtubules are fully disassembled when chromosomes reach the spindle poles (stage 7, Figure 4A T = 10 min, 4B T = 10 min top cell, T = 5 min bottom cell) and disassembly concludes when interpolar microtubules disappear. Phragmoplast formation begins in telophase, with a bright disk of microtubules located midway between chromosome masses (stage 8, Figure 4A T = 15 min, 4B T = 15 min top cell, T = 20 min bottom cell) and expands radially outward toward the cell cortex (stage 9, Figure 4 T = 35 min). Spindle disassembly is defined as the time from the initiation of anaphase (stage 6) to the appearance of the phragmoplast disk (stage 8). Phragmoplast expansion is defined as the time from appearance (stage 8) to the completion of outward expansion to the cell cortex (stage 9). The average spindle disassembly time for meiosis I is 16.2 ± 3.5 min and 14.6 ± 3.1 min for meiosis II (not statistically different, *p*-value > 0.05, *t*-test) (Figure 5D). Both meiosis I and II had a normal distribution of disassembly times (Figure 2B). Disassembly of the spindle showed a unique two-state process with a rate change near the end of disassembly. Beginning at the metaphase-to-anaphase transition, spindle length gradually decreased at a rate of 0.45 um/min for approximately 15 min, then exhibited a quick collapse (4.3 um/min) in the final 5 min (Figure 5E). The rate change was observed in all cells, and the difference was statistically significant (*p*-value < 0.001, *t*-test).

The phragmoplast expansion rate was constant at 0.85 um/min, and the total expansion time in meiosis I was 41.9 ± 11.8 min and 33.6 ± 3.8 min in meiosis II. The difference in time is statistically significant (*p* < 0.001, *t*-test) (Figure 5D). The variance in meiosis I phragmoplast expansion time is also greater than meiosis II, as can be seen in the broader distribution of times (Figure 5C, *p* = 0.001, f-test). Meiosis II cells are hemi-spherical, with spindles orienting along the longer length (blue line) and phragmoplasts expanding perpendicular to this dimension (red line) (Figure 5G). Average cell diameter in the phragmoplast dimension (red line) is shorter in meiosis II cells compared to meiosis I cells (28.4 ± 4.3 µm vs. 34.7± 8.0 µm vs., *p* < 0.01, *t*-test). Additionally, expansion time correlates with this length (R^2^ = 0.7592); meiosis I cells are plotted in black and meiosis II cells are plotted in gray (Figure 5F). A longer expansion time is required to build a larger phragmoplast to reach the cell cortex in the larger meiosis I cells.

### 2.4. Frequent Errors in Spindle Assembly Require Active Correction

**Figure 6:**
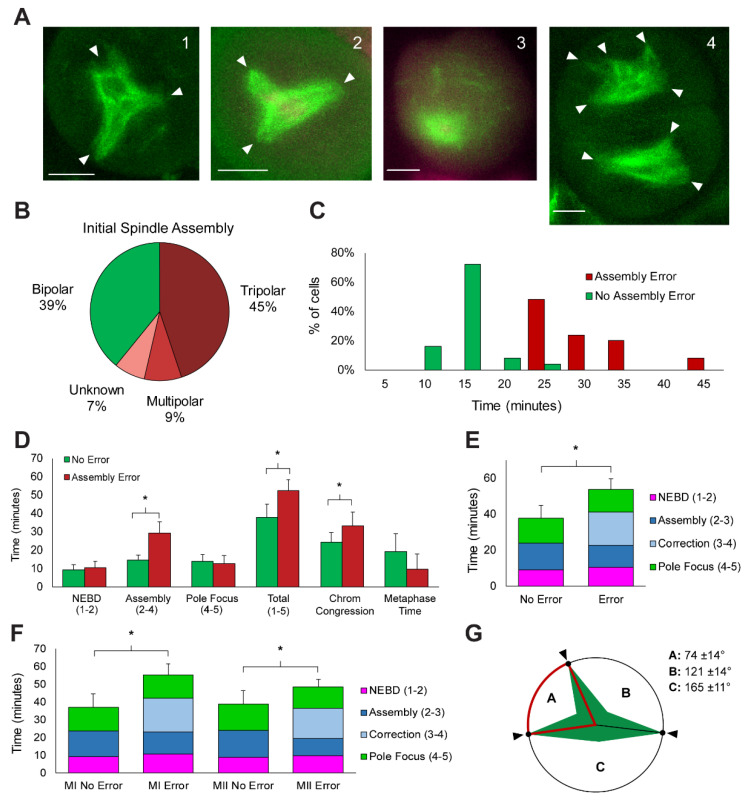
Characterization of spindle assembly errors and correction dynamics. (**A**) Examples of incorrect spindle morphology in meiosis I (panel 1–3) and meiosis II (panel 4): tripolar spindles (panel 1, 2, bottom cell in panel 4), unknown polarity (panel 3), and multipolar (top cell in panel 4). White arrows indicate poles. Scale bars: 10 µm. (**B**) Distribution of initial spindle morphologies: bipolar spindles (green) form only 39% of the time (*n* = 27/70), and the remaining assemblies contain errors (red), the most frequent being tripolar (*n* = 32/70). (**C**) Histogram of spindle assembly time; same data as Figure 2C but categorized by errors in assembly revealing two distinction populations. (**D**) Comparison of assembly dynamic parameters by error status. * denotes *p*-value < 0.001, *t*-test (**E**) Total assembly time in error and no error cells; breakdown by stage: NEBD (pink), initial assembly (dark blue), correction time (light blue), and pole focus (green). Error bars represent standard deviation. * denotes *p*-value < 0.001, *t*-test (**F**) Same stage breakdown analysis as displayed in (**E**) but separated by each population: meiosis I no error, meiosis I error, meiosis II no error, meiosis II error. * denotes *p*-value < 0.05, *t*-test (**G**) Correction of tripolar spindles by fusion of poles (black arrow). Fusion always occurred between poles separated by the smallest angle (A); average angle measurements displayed.

Live imaging of meiotic spindle assembly revealed that the process is error prone, with frequent instances of multi-polar spindles that were corrected into the bipolar form. After NEBD, initial spindle assembly yielded a variety of morphologies; example images are shown in Figure 6A, with white arrows marking spindle poles. Tripolar spindles were the most prevalent morphology, observed in 45% of cells (Figure 6A panel 1 and 2, Figure 6B, Supplemental Movies S6 and S7). The assembly of multipolar spindles (more than three) was observed in 9% of cells (Figure 6A panel 4, Figure 6B, Supplemental Movie S9) and spindles with no clear polarity were observed in 7% of cells (Figure 6A, panel 3, Figure 6B, Supplemental Movie S8). Spindles were initially formed in the correct bipolar morphology in only 39% of observed assemblies. Of the non-bipolar spindles, 85% (*n* = 39/45) corrected the initial assembly error during the period of observation. The remaining cells failed to correct the error and did not proceed to anaphase during observation.

The histogram plot of spindle assembly time (stage 2–4) showed a broad distribution of times that was not correlated to meiotic stage (Figure 2C). The distribution appears bimodal, with a peak around 15 min and a peak around 25 min, so we tested whether the dataset consists of two separate populations: a population of cells that initiated spindle assembly correctly and a population that had an error in initial spindle assembly. When plotted as these two populations (Figure 6C), the peaks are statistically different (*p*-value < 0.001, *t*-test). The average assembly time for spindles with no errors is 14.6 ± 2.9 min and assembly time for spindles with errors is 29.0 ± 6.3 min (Figure 6C). The variance in assembly time is also significantly greater in cells with errors (*p*-value < 0.001, f-test). Reanalyzing all of the dynamic parameters by the presence of errors (Figure 6D) showed that the increase in total assembly time (52.4 ± 6.0 min error vs. 37.7 ± 7.1 min no-error, *p*-value < 0.001, *t*-test) is due solely to increased time spent in stages 2–There was no difference in NEBD or pole focusing time (*p*-values > 0.05, *t*-test), but the time required to congress chromosomes was greater in cells with errors (33.1 ± 7.7 min vs. 24.1 ± 5.3 min, *p*-value < 0.001, *t*-test).

Analysis of the movies showed that the assembly process (stage 2–4) either proceeded directly from collapsed microtubules to a bipolar spindle in cells with no errors, or required a period of correction, where spindles reorganized from multipolar to bipolar spindles (Supplemental Movie S6). This period of correction time was, on average, 18.5 ± 4.6 min (light blue, Figure 6E), and it accounted for the increase in both assembly time (stage 2–4) and total time (stage 1–5) (*p*-value < 0.001) (Figure 6E). The initial assembly process (dark blue, Figure 6E) was statistically shorter in cells with an error (*p*-value = 0.02, *t*-test); cells that initiated an incorrect spindle spent, on average, 12.0 ± 5.2 min assembling the incorrect morphology then switched to correction. Spindles with no initial error spent 14.6 ± 2.9 min assembling the bipolar shape and required no correction time. The increase in total spindle assembly time was present in both meiosis I and II cells. Separating each population (MI error, MI no error, MII error, MII no error) revealed that total assembly time (stage 1–5) in both meiosis I and II was statistically greater in cells with errors than their counterparts without errors (*p*-value < 0.05, *t*-test) (Figure 6F). Assembly and correction time was longer in meiosis I than in meiosis II cells (29.9 ± 6.8 vs. 25.6± 1.3 min, *p*-value = 0.01, *t*-test).

**Figure 7:**
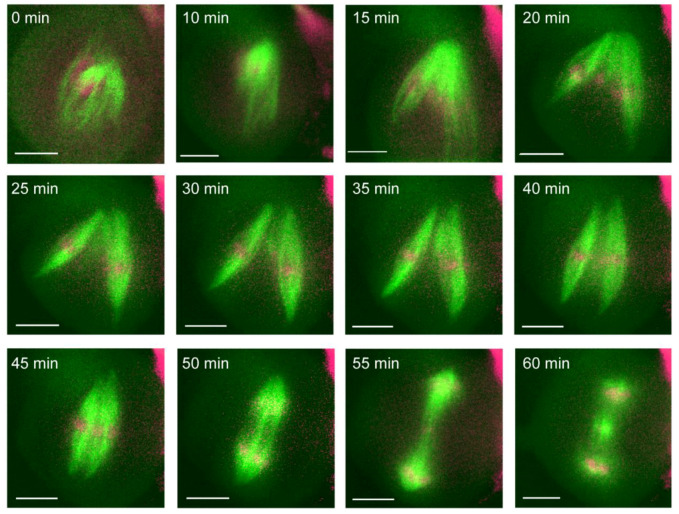
In vivo correction merging two bipolar spindles. Two bipolar spindles (green) were assembled in a single meiosis I cell (0–35 min). This error was corrected before anaphase (50 min) by merging the two spindles into a single spindle (35–45 min). After correction, the spindle successfully separated chromosomes (magenta) (50–60 min). Scale bars: 10 µm.

To correct assembly errors, multipolar spindles collapsed poles together to achieve a bipolar morphology (Supplemental Movies S6–S10). We measured the angle between tripolar spindle poles and tracked their fate during correction (Figure 6G). In all observed tripolar cells, the poles with the smallest separating angle were collapsed together (A in Figure 6G). The smallest angle (A) was, on average, 74 ± 14°, with the other angles averaging 121 ± 14° (B, intermediate angle) and 165 ± 11° (C, largest angle); all angles were statistically different from each other (*p*-value < 0.001, *t*-test). In one cell, we observed the assembly of two independent spindles that corrected into a single spindle (Figure 7). Initially two spindles were built around disparate chromosome masses (T = 0–20 min). The two spindles fused through a jack-knife action that brought them into parallel alignment (T = 25–45 min). After fusion into a single bipolar spindle, the cell proceeded to segregate chromosomes in anaphase (T = 50–55 min) and telophase (60 min).

## 3. Discussion

Acentrosomal spindle assembly relies on the self-organizational dynamics of microtubules and MAPs, as well as microtubule nucleation from non-centrosomal locations. Animal cells harness both cytoplasmic MTOCs, small asters of γ-tubulin visible in the cytoplasm before NEBD, and the nucleating potential of chromatin, regulated through the RanGTP and Chromosome Passenger Complex (CPC) pathways [23]. While RanGTP pathway members are conserved in plants [15,26] and CPC homologs have been recently identified [50], microtubule nucleation appears to be primarily driven by membranes [15]. Studies in maize [27], *Haemanthus* [28,29,51], *Arabidopsis* [32,41] and tobacco cultured cells [45,46] have shown microtubule localization and nucleation on the plasma membrane, the nuclear envelope, and other membrane bound surfaces. Nuclear envelope-based nucleation is particularly important in mitotic spindle assembly, as the γ-tubulin ring complex interacts with nuclear pore proteins, such as Nup88 [52] and Rae1 [51]. Microtubule foci on the nuclear envelope coalesce into polar caps before NEBD and organize a “prospindle” on the envelope surface [49,53,54]. After NEBD, the barrel-shaped mitotic plant spindle segregates chromosomes, and the phragmoplast appears in the former spindle midzone to direct cytokinesis [30,49].

Through live imaging of maize meiotic spindle assembly, we found that the process shares similarities with both plant mitotic assembly and female animal meiotic assembly. Previous live imaging studies of *Arabidopsis* meiosis revealed 11 identifiable landmarks, based on subcellular morphologies throughout the entire meiotic process [41]. Our analysis of live maize meiosis yielded nine identifiable stages, but located within a specific period from NEBD to phragmoplast expansion due to our increased temporal and spatial resolution. Identification of these stages allowed us to compare rates and dynamics to other systems. Similar to live studies of mitotic spindle assembly in maize [30,31], *Arabidopsis* [32], tobacco [33] and the moss *P. patens* [34], we observed a perinuclear ring of microtubules on the nuclear envelope surface (stage 1, Figure 1 and Figure 2A). However, we did not observe polar caps or prospindle-like structures; instead, our data indicate that homogenous perinuclear rings collapse into the nuclear space and self-organize into bipolar spindles, similar to female animals’ “slide and cluster” mechanism (Figure 1) [55]. Additionally, the diameter of the perinuclear ring correlates with spindle length (Figure 2E), suggesting this microtubule structure could help set spindle length.

The total time required to assemble a maize meiotic spindle (stage 1–5, ~45 min) was on the same time scale of minutes as maize and *Arabidopsis* mitosis (~40 min) [30,32], *P. patens* mitosis (10 min) [34] and *Arabidopsis* meiosis I and II (~60 min in metaphase I, >60 min in metaphase II) [41]. Animal meiotic spindle assembly occurs over many hours; studies on live human [8] and mouse oocytes [16,20,56] showed that spindle assembly occurs over ~16 h and 4–8 h, respectively. Chromosome congression showed a similar difference in timing, with 7 h required in human oocytes [8] and 3–8 h in mouse [16,56], but only 30 min in maize meiosis (Figure 3C).

Maize meiotic spindles focus their poles, unlike their mitotic counterparts [31], but in a similar way to female animal meiotic spindles [57]; this process takes ~13 min (Figure 2D). The focusing of plant meiotic poles is facilitated by the minus-end-directed kinesin-14, Dv1 in maize [58] and AtKIN14 in *Arabidopsis* [59,60], as plants lack most of the subunits of dynein [61] used by animal meiotic spindles [62]. After aligning chromosomes on the spindle, cells waited in metaphase for approximately 15 min before proceeding to anaphase. This hold time is considerably longer than observed in mitosis, where cells rapidly transition to anaphase [30,34], but it is significantly shorter than female animal meiotic cells, which hold in metaphase I for 1–2 h [8] and metaphase II for up to 6 h, until fertilization [63]. Animal meiosis is thought to have different metaphase-to-anaphase transition dynamics than mitosis due to the presence of a meiosis-specific cyclin B3 [64]. The observed maize meiotic hold time is longer than mitosis, but on a minutes, rather than hours, scale, so the difference here could be due to satisfaction of the spindle assembly checkpoint. Previous studies in fixed maize samples have shown that Mad2 localizes on kinetochores early in meiotic chromosome alignment [65], which activates the checkpoint and prevents transition to anaphase until all chromosomes are properly oriented [66]. The removal of Mad2 from the meiotic kinetochore may be longer given its structural differences [67], and accounts for this increased hold time.

Few studies have focused on the disassembly of the meiotic spindle following chromosome segregation. Live animal meiosis has shown a process that occurs over hours [20,56], and the *Arabidopsis* live meiosis study did not have the temporal resolution to determine disassembly time [41]. Live plant mitotic studies show a process that occurs over minutes, approximately 45 min in *Arabidopsis* [32] and 10–15 min in maize and *P. patens* [30,31,34]. In our observations, disassembly of the spindle in anaphase and telophase showed a unique two-state process, previously uncharacterized in other live systems. Beginning at the metaphase-to-anaphase transition, spindle length gradually decreases (stage 5–7) at a rate of 0.45 um/min then exhibits a quick collapse (4.3 um/min) to the phragmoplast (stage 8) (Figure 5E). These two rates are likely the result of microtubule stabilization by kinetochores during the initial disassembly, as the quick collapse occurs after chromosomes have reached the poles. The total disassembly time (6–8) of 16 min is similar to maize mitosis (10 min), but the rate change has not been observed (Figure 5B,D). The phragmoplast expansion rate was constant at 0.85um/min, faster than the previously measured maize mitotic rate of 0.2um/min [30] (Figure 5D,F).

The major defining feature of the observed spindle assembly process was the high incidence of errors in initial assembly, and the correction to bipolar form before anaphase (Figure 6A). Only 39% of cells initiated a bipolar spindle; the most predominant initial morphology was tripolar (45%) (Figure 6B). Live studies in human oocytes showed similarly high error rates, with less than 20% of cells initiating and maintaining stable bipolar spindles [8]. Other studies in live mouse oocytes showed that multiple small cytoplasmic asters produce multipolar spindles that require active fragmentation and coalescence of poles into the bipolar form [17]. Live studies of *Arabidopsis* meiosis did not report multipolar spindles [41]. However, the *Arabidopsis* study was focused more broadly on meiosis landmarks, where data were collected with 3D z-step acquisitions of >8µm that lacked the spatial resolution to identify these events. Studies using fixed samples found a similar occurrence of multipolar meiotic spindles in rice, maize, tomato, *Arabidopsis* [18], *C. elegans* [68] and mice [16]. In rice, multipolar meiosis I spindles transitioned to bipolar form through the assistance of OsMTOPVIB [18], a protein essential for recombination and double-strand break formation [69,70]. Interestingly, we found that error and correction rates, as well as most dynamics of assembly and disassembly, were indistinguishable between meiosis I and meiosis II (Figure 2Figure 3 and Figure 5). The similarity in meiosis I and II dynamics suggests that regulation of these processes may not be exclusive to meiosis-I-specific activities, including DSB formation and recombination.

While there are no statistical differences between meiosis I and II dynamics (except phragmoplast expansion time), many parameters were statistically different between cells with and without assembly errors (Figure 6). Initiating an incorrect morphology significantly increased total assembly time due to the required correction time. Likewise, chromosome alignment time was increased, but time spent in metaphase was not different, suggesting an internally regulated metaphase timing rather than the previously suggested external synchronization of meiotic entry [71]. The time spent building the initial spindle was statistically shorter in cells with an incorrect morphology compared to those with bipolar spindles (Figure 6E), suggesting active assessment of spindle shape and a switch to correction. The mechanism of spindle correction was the same in all observed cells; in multipolar spindles, poles separated by the smallest angle coalesced into a single pole (Figure 6G). The most likely mechanism for sensing and correcting these errors is the spindle assembly checkpoint, a surveillance mechanism known to monitor the attachment of chromosomes to the spindle in both mitosis and meiosis [72]. RNAi screens in *Drosophila* S2 cells and cancer cells have found that spindle assembly checkpoint proteins suppress multipolar spindles [73]. Additionally, the spindle assembly checkpoint actives in response to multipolar spindles, demonstrating a sensitivity to spindle morphology [73,74]. Several of the major effector proteins in the spindle assembly checkpoint have been identified in maize, including Mad2 [65], Bub1 and 3 [75], and Aurora B kinase [76].

We also observed the fusion of two spindles into a single bipolar spindle (Figure 7), a phenomenon previously observed in cultured mitotic neuroblasts [77] and produced by micromanipulation in cell extracts [78]. In vitro studies have shown that spindles will form around masses of chromosomes, separated by sufficient distance (>10 µm) [77,78,79]. These independent spindles can fuse into a single spindle via a fusion of proximal poles and a jackknifing action that brings distal poles together [77,78]. We observed a similar jackknifing mechanism of spindle fusion (Figure 7), but a unique mechanism must be employed as maize lacks dynein [61], which was previously found to be required for this action [78].

Live imaging allows the continuous monitoring of individual cells to capture data about dynamic processes, not otherwise measurable in fixed specimens. Here, we analyzed live spindle assembly and disassembly in maize meiotic cells, and confirmed previous reports of an error-prone process [18], as well as demonstrating that individual spindles actively correct these errors through structural rearrangements before proceeding to anaphase. The dynamics of spindle assembly are indistinguishable between meiosis I and II, suggesting a general regulation mechanism for acentrosomal assembly, not specific to early meiotic events as previously thought. Future studies will investigate potential regulatory pathways governing spindle assembly, including RanGTP, CPC and the spindle assembly checkpoint. Understanding the regulation of spindle assembly and the resulting chromosome segregation is particularly important in maize. The transmission of traits and viability of gametes are critical for the production of this major agricultural crop, and ensuring accuracy in these processes could support enhanced breeding efforts [80] to sustain growing worldwide food needs [81,82].

## 4. Materials and Methods

### 4.1. Maize Lines and Genotyping

A transgenic maize line (*Zea mays* ssp. mays) containing an N-terminal fusion of β-tubulin (β-TUB1) with CFP was generated by the laboratory of Anne Sylvester (University of Wyoming, Laramie, WY, USA). Transgenic plant leaf tissue was genotyped using a CTAB DNA extraction protocol (Clarke, 2009) and primers that spanned the CFP-β-TUB1 transgene (forward, anneal in CFP: 5′-GGAGTACAACTACATCAGCCACAACGTC.; reverse, anneal in β-TUB1: 5′-CCGGACTGACCGAAGACGAAGTTGT). All chemicals and reagents were purchased from Sigma Aldrich (St. Louis, MO, USA) unless otherwise noted.

### 4.2. Live Imaging

Male meiotic cells were harvested from immature tassels as previously described [39]. Isolated meiotic cells were cultured in medium [39] containing 2 µM STYO12 Green DNA dye to label chromosomes (Invitrogen Molecular Probes, Eugene, OR, USA). Cells were staged for meiotic progress, loaded onto coverslips (Cornings, Corning, NY, USA) coated with 1 mg/mL poly-L-lysine, and sealed onto slides (Fisher Scientific, Waltham, MA, USA) as previously described (Nannas and Dawe, 2016). Cells were imaged at 21 °C on a Zeiss Axio Imager.M1 and a Zeiss Axio Observer 7 Marianas fluorescence microscope with a 63×/1.4 NA Plan-APO Chromat oil objective. Images were collected every 3–7 min in three dimensions over a 20 µm Z-range with 1 µm Z-steps with 50 ms exposure for CFP and 30 ms exposure for SYTO12 and 2 × 2 binning. Sample size was sufficient for Student’s *t*-test used in statistical analysis described in the text.

### 4.3. Image Analysis

Images were analyzed using Slidebook software (Intelligent Imaging Innovations, Denver, CO, USA). Meiotic stages and processes were assigned based on identifying features: nuclear envelope breakdown time was measured as the first time point showing deformation of the spherical nuclear envelope to the complete collapse of microtubules into the nuclear space (stage 2). Spindle assembly time was measured as the time from the end of nuclear envelope breakdown until the emergence of bipolar spindle (stage 4). Spindle assembly status was determined by pole number; correct spindles contained 2 poles, while spindles with greater or fewer than 2 poles were considered incorrect. Poles were identified using Slidebook’s 4-Dimensional Viewer function (3-dimensional visualization through time). Angles between spindle poles were measured using the angle measurement tool. Spindle disassembly time was measured as the time from the first anaphase time point (stage 6) until the appearance of the phragmoplast (stage 8). Phragmoplast expansion time was measured as the time required for the phragmoplast to reach the cell cortex (stage 9). The perinuclear microtubule ring, spindle and phragmoplast were identified as objects by thresholding at approximately 50% above background CFP signal as previously described [38]. Object statistics were extracted using Slidebook, and the perinuclear ring diameter, spindle length, and phragmoplast diameter were measured as the longest chord within the object (distance between the two furthest pixels within the object). Student’s *t*-tests and f-tests were used to analyze differences in spatial and temporal measurements.

## Data Availability

Not applicable.

## Supplementary Materials

The following supporting information can be downloaded at: https://www.mdpi.com/article/10.3390/ijms23084293/s1.
